# News and Notes

**Published:** 1912-08

**Authors:** 


### NEWS AND NOTES

### WILLIAM EDWARD FITCH, M. D., M. R. C., U. S. A.

President Taft, with the confirmation of the Senate, on July thirtieth commissioned William Edward Fitch, M. D., (Editor of Pediatrics, New York City) to be a First Lieutenant in the Medical Reserve Corps of the Army of the United States, with rank dating from uly 3rd, 1912.

---

### TRAINED NURSE—PHILIPPINE SERVICE, OCTOBER 16, 1912.

The United States Civil Service Commission announces that the examination for trained nurse in the Isthmian Canal and the Indian services will be held on October 16, 1912, as scheduled, but that the announcement of the examination for this position in the Philippine service is canceled because of advice from the Bureau of Insular Affairs that future vacancies in this position in the Philppne service will likely be filled by Filipino women. Issued Aug. 14, 1912.

---

### EXAMINATION OF DENTISTS FOR THE U. S. ARMY.

The Surgeon General of the Army announces that examinations for the appointment of Acting Dental Surgeons will be held at Fort Slocum, New York; Columbus Barracks, Ohio; Jefferson Barracks, Missouri; Fort Logan, Colorado, and Fort McDowell, California, on Monday, October 7, 1912.

Application blanks and full information concerning these examinations can be procured by addressing the "Surgeon General, U. S. Army, Washington, D. C."

The essential requirements to securing an invitation are that the applicant shall be a citizen of the United States, shall be between 21 and 27 years of age, a graduate of a dental school legally authorized to confer the degree of D. D. S. and shall be of good moral character and habits.

Acting Dental Surgeons are employed under a three years' contract, at the rate of $150.00 per month. They are entitled to traveling allowances in obeying their first orders, in changing stations, and in returning to their homes at termination of service. They also have the privilege of purchasing certain supplies at the Army commissary. After three years' service, if found qualified, they are promoted to the grade of dental surgeon with the rank of first lieutenant, and receive thereafter the pay and allowances appertaining to that rank.

In order to perfect all necessary arrangements for examination, applications must be in the possession of the Surgeon General at least two weeks before the date of examination. Early attention is therefore enjoined upon all intending applicants. There is at present a large number of vacancies to be filled.

---

### MEDICAL PRACTICE BILL.

### A Bill

To be entitled an Act to establish a composite Board of Medical Examiners for the State of Georgia; to define its duties and powers; to protect the people from illegal and unqualified practitioners of medicine and surgery; to define the standing of a medical college; to regulate the issuing and recording of licenses; to define what is considered the practice of medicine; to fix fee for license; to provide for the revocation of license; to require a standard of preliminary education of applicants; to prescribe penalties for the violation of this Act; and for other purposes.

### Section 1.

Be it enacted by the General Assembly of Georgia, and it is hereby enacted by the authority of the same, that a Board is hereby established, to be known by the name and style of the State Board of Medical Examiners. Said Board shall be composed of eight practicing physicians of integrity and ability, who shall be residents of, and have been duly licensed to practcie medicine in this State, and who shall have graduated from reputable medical schools and have been engaged in the active practice of their profession within this State for at least a period of five years; but none of them shall be connected in any way with any medical college. Said Board shall perform such duties, and possess and exercise such powers, relative to the protection of the public health, and the control and regulation of the practice of medicine in this State as shall be in this Act prescribed and conferred upon it.

SECTION 2.

Be it further enacted, That the Governor shall within thirty days after the passage of this Act, appoint eight physicians, who shall possess the qualificationh specified in Section 1 of this Act, to constitute the members of this Board. Five members of this Board shall be regular physicians, two shall be Eclectic physicians, one shall be a Homeopathic physician—all to be appointed by the Governor from the latest lists of names furnished by the respective State Medical Association. The successor of each member shall be an appointee in the same manner. Said members shall be so classified by the Governor that the term of office of two shall expire in one year, two in two, two in three, and two in four years from the date of appointment. Annually thereafter the Governor shall appoint two members, each of whom shall serve for a term of four years; and these appointments shall be made so as to preserve the original ratio of Regulars, Eclectics and Homeopaths, respectively. Any vacancy that may occur in said Board, in consequence of death, resignation, removal from the State, or from other cause, shall be filled for the unexpired term by the Governor in the same manner. A majority of the Board shall constitute a quorum.

SECTION 3.

Be it further enacted, That immediately and before entering upon the duties of said office, the members of said Board shall tak the constitutional oath of office and shall file the same in the office of the Governor of the State, who upon receiving the said oath of office, shall issue to each member a certificate of appointment.

SECTION 4.

Be it further enacted, That immediately after the appointment and qualifications of said members, said Board shall meet and organize. Said Board shall elect a president and vice-president from the membership, and a secretary-treasurer who shall be a physician and who, by virtue of his office, shall be an ex-officio member of the Board, whose term of office shall be governed by the pleasure of the Board and whose salary shall be fixed and paid by the Board. All expenses of the Board shall be paid out of the funds collected by the Board. Said Board shall hold two regular meetings in each year. One meeting shall be held in May or June at such time as suits the conveniience of graduates of medical colleges in Atlanta and Augusta; the other meetings shall be held on the second Tuesday in October in the State Capitol. Call meetings may be held at the discretion of the president. The regular meetings shall be held at the Capitol building in Atlanta, and in Augusta. Said Board shall adopt a seal, which must be affixed to all licenses issued by them. They shall from time to time adopt such rules and regulations as they may deem necessary for the performance of their duties, and shall examine and pass upon the qualifications of applicants for the practice of medicine in this State, as herein prescribed.

SECTION 5.

Be it further enacted, That any person wishing to obtain the right to practice medicine in this State, who has not heretofore been licensed so to do, shall, before it shall be lawful for him to practice medicine in this State, make application to the Board, through the secretary-treasurer thereof, upon such form and in such manner as shall be adopted and prescribed by the Board, and obtain from the Board a license so to do. Unless each person shall have obtained a license as aforesaid, it shall be unlawful for him to practice in this State, and if he shall practice medicine in this State without first having obtained such a license, he shall be deemed to have violated the provisions of this Act. All applicants for a license to practice medicine or for a renewal of any such license which has been revoked, shall furnish the Board, with evidence of good, moral character. Applicants from candidates to practice medicine or surgery in any of the branches shall be accompanied with proof that the applicant is a graduate of a legally incorporated medical college or institution in good standing with the Board. The Board shall have the power to revoke the certificate granted to any applicant who makes any misstatement of any material fact in his application for examination. Each applicant shall name in his application the system of practice he proposes to follow, and no person shall use the name of any system unless he holds a certificate from the Board.

SECTION 6.

Be it further enacted, That before any person who obtains a certificate from said Board may lawfully practice medicine and surgery in this State, he shall cause the said certificate to be recorded in the office of the Clerk of the Superior Court of the county in which he resides. The certificate shall be recorded by the Clerk in a book kept for that purpose. It shall be indexed in the name of the person to whom the certificate is granted. The Clerk's fee for recording the certificate shall be the same as for recording a deed. The Clerk shall make a report to the Secretary of the Board on the 31st of December of each year, of all certificates registered with him. Each applicant receiving a certificate from the Board shall cause same to be registered within thirty days.

SECTION 7.

Be it further enacted, That said Board shall be empowered by this Act to pass upon the good standing and reputability of any medical college. Only such medical colleges will be considered in good standing as possess a full and complete faculty for the teaching of medicine, surgery, and obstetrics, in all their branches, afford their students adequate clinical and hospital facilities, require attendance upon at least 80 per cent. of each course of instruction, give four graded courses of instruction, the aggregate of which amounts to at least one hundred and twenty weeks, exclusive of holidays, of at least forty hours each week; that require at least forty-two months to have elapsed between the beginning of the student's first course of medical lectures and the date of his graduation, each session composed of twenty-nine weeks of actual instruction, with at least forty per cent. of laboratory instruction in the first and second years, and a minimum of thirty-five per cent. of clinical work in the third and fourth years; that require an average grade in each course of instruction of at least seventy-five per cent. in examination as a condition of graduation; that fulfill all their published promises, requirements and other claims respecting advantages to their students and the course of instruction; that enact a preliminary educational requirement equal to that specified by this Act; that require students to furnish testimonials of good moral standing; and that give advance standing only on cards from accredited medical colleges. Students must have attended at least ninety per cent. of the course in the last year of the college from which diploma is presented. In determining the reputability of the medical college, the right to investigate and make a personal inspection of the same is hereby authorized.

SECTION 8.

Be it further enacted, That beginning with the session of 1912 and 1913, each medical school or college in good standing with the Board shall have a minimum preliminary educational requirement of twelve Carnegie units.

SECTION 9.

Be it further enacted, That in the discretion of the secretary-treasurer of said Board, with the approval of the president, he may issue temporary license to an applicant, which shall have the same force and effect of a permanent license until the next regular meeting of the Board, when said license shall become void. Said license shall not be recorded.

SECTION 10.

Be it further enacted, That examination of applicants for license to practice medicine shall be made by said Board according to the methods deemed by it to be the most practical and expeditious to test the applicant's qualifications. Where the Board requires the examination to be in writing, each applicant shall be designated by a number instead of his name, so that his identity shall not be disclosed to the members of the Board until after the examination papers are graded. Examination shall be on the following subjects: Anatomy, physiology, chemistry, pathology, surgery, obstetrics, practice, pediatrics and other subjects in the discretion of the Board. Students may be examined on anatomy, physiology, chemistry, materia, histology, bacteriology and embryology and given credit for same after having completed two years of instruction.

SECTION 11.

Be it further enacted, That there shall be paid to the secretary-treasurer of said Board by each applicant for a license by examination, a fee of twenty dollars which shall accompany the application. The same fee shall be charged for issuing a temporary license, which includes fee for examination for permanent license; and a fee of fifty dollars shall be charged for issuing a license by reciprocity. No part of any fee is returnable under any circumstances. Nor shall this Act be construed as affecting or changing, in any way, laws in reference to license tax to be paid by physicians and surgeons.

SECTION 12.

Be it further enacted, That said Board shall have authority to administer oaths, to summon witnesses and to take testimony in all matters relating to its duties. Said Board shall issue licenses to practice medicine to all persons who shall furnish satisfactory evidence of attainments and qualifications under the provisions of this Act, and the rules and regulations of the Board. Such license shall be signed by the president and attested by the secretary-treasurer of the Board under its adopted seal, and it shall give absolute authority to the persons to whom it is issued to practice medicine in this State. It shall be the duty of the secretary-treasurer, under the direction of the Board, personally or by deputy, to aid the solicitors of the State in the enforcement of this Act, and in the prosecution of all persons charged with violation of any of its provisions.

SECTION 13.

Be it further enacted, That said Board may refuse to grant, or may revoke a license to practice medicine in this State, or may cause a licentiate's name to be removed from the records in the office of any Clerk of Court in the State on the following grounds, to-wit:

The employment of fraud or deception in applying for license or in passing the examination provided for in this Act; conviction of crime involving moral turpitude; the practice of medicine under a false or assumed name, or the impersonation of another practitioner of a like or different name; habitual intemperance in the use of ardent spirits, narcotics, or stimulants to such an extent as to incapacitate him for the performance of professional duties; the procuring or aiding or abetting in procuring a criminal abortion; the obtaining of a fee on representation that a manifestly incurable disease can be permanently cured; causing the publication and circulation of an advertisement of any medicine by means whereby the monthly periods of women can be regulated, or the menses, if suppressed, can be re-established; causing the publication and circulation of an advertisement relative to any disease of the sexual organs. Said Board may at any time within two years from the refusal or revocation of a license or cancellation of a registration under this Section, by a majority vote, issue a new license or grant a license to the person affected restoring or conferring all the rights and privileges of and pertaining to the practice of medicine as defined and regulated by this Act. Any person to whom such rights and privileges have been so restored shall pay to the secretary-treasurer a fee of twenty dollars on the issuance of a new license.

SECTION 14.

Be it further enacted, That the terms "practice of medicine," "to practice medicine," "practicing medicine," and "practice of medicine," as used in this Act, are hereby defined to mean holding oneself out to the public as being engaged within this State in the diagnosis or treatment of diseases or injuries of human beings; or the suggestion, recommendation or prescribing of any form of treatment for the intended palliation, relief or cure of any physical or mental ailment of any person, with the intention of receiving therefor, either directly or indirectly, any fee, gift or compensation whatever, or the maintenence of an office for the reception, examination and treatment of any person suffering from disease or injury of body or mind; or attaching the title "M. D.," surgeon, doctor or any other word or abbreviation to his name, indicative that such person is engaged in the treatment or diagnosis of diseases or injuries of human beings. If any person shall hold himself out to the public as being engaged within this State in the diagnosis or treatment of diseases or injuries of human beings; or shall suggest, recommend or prescribe any form of treatment for the palliation, relief or cure of any physical or mental ailment of any person with the intention of receiving therefor, either directly or indirectly, any fee, gift or compensation whatsoever; or shall maintain an office for the reception, examination or treatment of diseased or injured human beings; or shall attach the title "M. D.," surgeon, doctor or any other word or abbreviation to his name indicative that he is engaged in this State in the treatment of diseased or injured human beings; and shall not in any of these cases, then possess, in full force and virtue, a valid license to practice medicine under the laws of this State, he shall be deemed to be practicing medicine without complying with the provisions of this Act and violation thereof. Nothing in this Act shall be construed to prohibit gratuitious service in cases of emergency, nor the practice of the religious tenets or general beliefs of any church whatsoever, nor prescribing medicine or administering drugs. Nor shall it apply to commissioned surgeons of the United States Army, Navy, or Public Health and Marine Hospital Service, while so engaged, nor to regular licensed physicians called from other States or Territories to attend to special cases in this State, nor to the practice of dentistry, nor to mid-wives or nurses.

SECTION 16.

Be it further enacted, That any person guilty of practicing medicine in this State without complying with the provisions of this Act, or any person who shall violate the provisions of this Act, shall be deemed guilty of a misdemeanor, and upon conviction thereof, shall be punished as for a misdemeanor according to Section 1039 of the Penal Code of Georgia, Volume III. Any person presenting or attempting to give as his own the diploma or certificate or credentials of another, or who shall give false or forged evidence of any kind to the Board, or any member thereof, in connection with an application for a license to practice medicine, or shall practice medicine under a false or assumed name, or shall falsely impersonate another practitioner of a like or different name, shall be deemed guilty of a felony, and upon conviction thereof shall be punished by a fine of not less than $500.00 or more than $1,000.00, or by a term from two to five years in the penitentiary in this State, subject to the provisions of Section 1039 of the Penal Code of Georgia.

SECTION 17.

Be it further enacted, That on investigation of an applicant's credentials from another State Board, this Board may, when convinced that he is qualified to practice medicine, grant him a license without further examination.

Be it further enacted, That all laws and parts of laws in conflict with this Act be, and the same are, hereby repealed. This law does not apply to Osteopaths so long as they do not prescribe, dispense or administer drugs.

---

### AMERICAN PROCTOLOGIC SOCIETY.

### FOURTEENTH ANNUAL MEETING, HELD AT ATLANTIC CITY, N. J., JUNE 3 and 4, 1912.

*(Continued from Last Issue.)*

*Post-Operative Care of Rectal Cases.—By Wm. M. Beach, M. D., of Pittsburgh, Pa.*

Success in the solution of proctologic problems is measured by the degree of perfection in the restoration of functional conditions involved; we must remove the disease, but it is quite as important to have a cure to vouchsafe to our patients, perfect function.

Post-operative developments that need our attention are:

1. The disturbance of the nervous system; 2. The disturbance of the vascular system; 3. Digestive derangement; 4 Local conditions.

Post-operative neuroses manifest by (a) shock, (b) nervousness, (c) pain; (d) sphincteralgia, (e) retention of urine.

Vascular aberrations are shown by (a) hemorrhage, (b) infection.

Gastro-intestinal derangements are (a) nausea, (b) constipation, (c) ampullar impaction.

The local care of wounds should be inspected daily by the operator.

If patients are given proper post-operative care, their dread of radical cures would quickly subside, and rectal surgeons would escape untoward sequelae they may be compelled to record.

---

*Patulous Anus: Its Clinical Significance.—By Alfred J. Zoebel, M. D., of San Francisco, Cal.*

The condition of patulous anus results from an abnormal loss of tone in the sphincter muscles, which may be due to either a fault intrinsically within the muscle, or to some disturbance in its nerve supply. When purely muscular the cause may be a direct injury to the muscle; an infiltration by a malignant or a syphilitic growth; a participation in a general muscular weakness; or the presence of a foreign body in the rectum which prevents the muscle from completely contracting. When the nerve supply to the sphincters is at fault, the causative lesion may be either central or peripheral.

Complete fecal incontinence does not necessarily follow when the anus becomes patulous. The external sphincter, when but slightly affected, sometimes is assisted in performing its function by an extra effort of the will and through augmenting the muscle's action by strongly contracting the Glutei muscles and bringing them together.

A brief report of a few very interesting cases of patulous anus is given to illustrate the different causes of this condition, among them being a case of infiltration of the sphincters by a carcinomatous growth low down in the rectum; a case, the result of pederastic practices; a case, the result of a participation in the general alcoholic neuritis; cases where it occurred in low intussusception of the bowel in children; and two cases where it appeared as one of the early signs of Locomotor Ataxia.

---

*The Surgery of Colonic Constipation—A Report of Thirteen Cases.—By Louis J. Hirschman, M. D., of Detroitfi Mich.*

After presenting the histories, radiographs and reports of operative treatment of thirteen cases of obstipation due to colonic obstruction, dilatation, stricture and adhesions, Dr. Hirschman has formulated several principles in dealing with his cases requiring colonic surgery. They are epitomized in the following conclusions:

1. Most cases of chronic constipation are colonic in origin and many are obstructive in type.

2. Many cases of so-called chronic constipation are therefore really colonic obstipation.

3. Many cases of colonic obstipation suffer from dilatation of the colon with or without ptosis.

4. Radiography is a most vital necessity in the diagnosis of all cases of chronic interference with bowel function. Its negative value may be greater than its positive.

5. A chronically, over-distended colon, whether adherent or not, never again becomes a normally functionating bowel.

6. Intestinal adhesions usually tend to recur in encreased intensity and adhesions only cause symptoms when put under stress or tension.

7. Prevention of tension in physiologic rest to the affected organ and colonic rest is obtained only by colectomy, colostomy, or exclusion.

8. Colectomy as advocated by Lane is an operation seldom advisable and has many obvious objections from the standpoint of patient and physician. It is too grave a procedure to be undertaken except in the most aggravated cases.

9. Strictures, neoplasms, and other obstructions should be removed by excision of the diseased tissue and lateral anastomosis of the bowel.

10. Exclusion by ileo-colostomy is safe, easy to perform, and most satisfactory in the restoration of normal peristalsis and consequently normal health.

11. Results speak more eloquently than words. After an experience with nearly fifty cases requiring exclusion or resection of the colon for obstructive constipation with but one failure, I feel fully justified in recommending it to your careful consideration in all cases of aggravated colonic obstipation whether congenital, post-operative, or dependent upon some mechanical obstruction or narrowing of the bowel.

---

*The Roentgenologic Method of Examining Cases of Constipation and Obstipation—A Method of Visualization of Abdominal Lesions of the Intestinal Tract.—By Arthur F. Holding, M. D.,* of *New York City, New York.*

The author noted that current text-books on diagnosis written by eminent authorities are still copying cuts which were drawn by some artist rather than by an anatomist. Let us hope that the striking proof furnished (by the X-rays) of the fallacy of such teaching will be effective, and perhaps not one of the least results will be to cause true illustrations to be placed before our students' eyes.

The normal position of the colon and the parts of the intestine that can ordinarily be visualized by means of bismuth ingesta and the X-rays, are:

(1) The first portion of the duodenum; (2) the jejunum; (3) the ileum; (4) all parts of the colon; in some cases the second and third portions of the duodenum and the appendix can be visualized.

The accuracy, reliability and interpretation of findings by this method, however, may well receive our careful attention.

In the first place, this method does not cause gastro-intestinal symptoms, such as nausea, vomiting, diarrhea, constipation, gastro-intestinal or general symptoms, other than are present when buttermilk alone is ingested; it is, therefore, logical to assume that the buttermilk-bismuth mixture does not irritate the mucous membrane and gives a true picture of the motor activities of the patient's intestines.

By fluoroscopy and by radiography in the erect or prone positions, or both, an accurate outline of the lumen of the tract can be obtained, especially where there is any obstruction to the onward progress of the intestinal contents. The individual peristaltic waves can be accurately registered on a special photographic emulsion that is far more sensitive than the human retina and the progress of the peristaltic waves can thus be seen functionating under normal conditions, the patient and his abdominal contents not relaxed by a general anaesthetic; the secretions and motility not disturbed by the presence of an irritating foreign body, such as a stomach tube; the conclusion not based on inference deducted from chemical reactions of juices obtained by abnormal and irritating methods. The organic outline obtained in X-Ray plates is even more conclusive and reliable than the information obtained by the sense of touch whether that be applied over the intact abdominal wall or to the viscera laid bare by an exploratory incision. The radiographic emulsion and the retina are the two most sensitive methods of observation possessed by man, far outranking in their acuteness either the drum, membrane or the sense of touch. It has been contended that the abdominal operation was more accurate than an X-Ray examination, because it laid bare the "naked truth," the finality of this argument is based more on the sound of the words than in fact, as anyone knows who has had an opportunity to use both methods on the same case.

On the other hand, there is great danger of arriving at wrong conclusions in using the X-Ray method, especially when the examination is based on too few plates or is only an examination of a suspected part of the 30 odd feet of intestinal canal.

We must not let seniority interfere with our recognition of the superiority of methods employed by us for diagnosis. No progressive proctologist or surgeon should depend on any one method but should use them all in examining cases, and in obscure cases he should not hesitate to insist upon supplementing the more common methods of examination with a radiologic examination, regardless of the expense involved.

The various lesions and conditions that have been successfully shown by the X-Ray method are: atonic and spastic constipation; congenital anomalies of the tract, such as non-rotation of the cecum and narrowing or insufficiency of the ileo-cecal valve; adhesions, kinks, with or without adhesions, (including Lane's); ulcers, tumors within the canal and tumors pressing up the intestines from without.

It must be borne in mind that a palpable tumor disappearing after the administration of an enema or a cathartic, even if followed by improvement in the patient's condition, is not proof that the tumor was feces.

The Roentgenologic method of clarifying difficult conditions present in patients will no doubt be gladly welcomed and widely utilized by surgeons, who, as a class, deserve our greatest respect and admiration for their courage in attacking many ordinarily undiagnosible conditions by cutting boldly into the abdomen and making their diagnosis by inspection and thereupon instituting impromptu surgical procedures in order to correct the conditions found. Many times the condition found within the abdomen is entirely different from that which was expected. When these difficult procedures can be accurately predetermined; when much time (previously lost exploring the abdomen) can be saved; when the duration of the patient's anaesthesia can be proportionately shortened; when the surgeon will be saved the tremendous nervous strain and responsibility of emergency decisions and procedures; the surgeon must recognize that his operative statistics will necessarily be better, his patients are going to recover quicker, and more of them, and finally, the years of a surgeon's own life and usefulness will be increased.

The only great drawback to the general adoption of this method is its necessarily great expense.

*(To Be Continued.)*

